# Ohio State Dental Society, Columbus, December 2d., 1874

**Published:** 1875-01

**Authors:** F. W. Sage


### Proceedings of Societies.

### OHIO STATE DENTAL SOCIETY, COLUMBUS, DECEMBER 2d., 1874.

REPORT OF DISCUSSIONS BY F. W. SAGE, D. D. S.

WEDNESDAY AFTERNOON, DEC. 3d.

The President, Dr. H. A. Smith, announced the first subject for discussion: The Preservation of the Pulps of Teeth. Subdivisions as follows:

(*a.*) Systemic conditions modifying treatment and influencing its results.

(*b.*) The least injurious and yet efficient agents for covering and protecting the dental pulp and likewise promoting the formation of secondary dentine.

(*c.*) Causes of failure.

Dr. C. R. Butler, of Cleveland, opened the discussion, and said: It would seem that taking the first and second subdivisions of the subject presented for discussion, under consideration, they naturally coalesce. We can easily conceive of systemic conditions which would be very unfavorable to the accomplishment of anything like satisfactory results. Under many circumstances we could not reasonably expect good results. In cases where the pulp is even very slightly exposed the chances for its preservation are not equal to the chances where the organization is good. In cases where the constitution of the individual has become weakened and prostrated from any cause, we would hardly expect the highest degree of success. There are a variety of modifying phases which are to be admitte.l and considered. In some cases we may almost abuse the pulp tissue and yet it persistently retains its vitality. Cut away a portion of it and even apply escharotics, as is the mistaken practice of some; (and I have known cases where arsenic has been applied) and yet the pulp has appeared almost entirely unaffected by the abuse. On the other hand I have seen cases where the least exposure has been sufficient to destroy the nerve pulp; so that we can only expect to arrive at anything like an intelligent course of practice to be pursued by careful observation of the structure of tissues involved—the temperament and organization of the patient—to govern us in our efforts to restore to health and preserve the pulp. As to materials to be employed, there is a great variety. Such are to be recommended as can best be adapted to the part which it is desired to protect and which are non-conducting and do not cause irritation. Perhaps one of the best, under ordinary circumstances, is Hill's stopping or gutta-percha. The use of the plastic materials is of course well known to us all, but their effect upon the pulp is not so well understood.

Some positively deny that all pulps are saved intact under these coverings; others assert that all or nearly all die. I am not prepared to say that I concur in the views or opinions of either of these positivists.

We cited a case of a young lady who at the age of fourteen had pulps capped in two inferior molars. Hill's stopping being used. Upon examination five years afterward, the pulps were found to be in good state of preservation, secondary dentine having been deposited. The teeth were of delicate organization, white and translucent. He discovered their condition after removing the temporary fillings. Filled them without farther treatment with gold. He continued: "I might cite other cases where Hill's stopping had been used successfully. Have seen plastic caps used in some cases with success, in others, unsuccessfully. It is to be remembered that the tooth is not a soft tissue—that it is not endowed with the highest vitality.

Dr. Watt was called, and responded as follows:

While I agree that the tooth is not endowed with the highest degree of vitality, I will say that the pulp has a vigorous circulation through all its capillaries and ramifications. It is unfortunate that there is such a prejudice against all efforts to preserve an aching pulp in the popular mind. It is mortify ing to us to know that a certain class of practitioners are easily influenced by the opinions and expressed wishes of the of the patient.

I suppose there is no more prevalent impression, erroneous impression, than that which obtains in the public mind that if the "nerve" be extirpated the tooth can not ache.

An individual comes to you with a fragment of a tooth sticking in the gum and requests to have the "nerve" killed. I suppose it is that education brought about through the agency of daily papers. For twenty-two or three years an advertisement has appeared in a Cincinnati daily, announcing "Nerves Destroyed," etc. Now since the nerve is endowed with high vitality we have a foundation to base an opinion upon, in considering whether or not it is practicable to preserve. the vitality after exposure. Once exposed it is in a very precarious condition. The great difficulty is a lack of faith and confidence on the part of the dentist. Most of us become too easily discouraged because of failure after all our care in treatment.

Hence, we easily come to decide that when a nerve becomes exposed we must annihilate it. I believe that some of you who have read the journals for years past, will remember that I once published my opinion that there is no more necessity for the indiscriminate destruction of the pulps of aching teeth than there is for amputation in all cases of compound fracture of limbs. You should take into consideration peculiarities of temperament, hereditary peculiarities, etc. If there is a scrofulous diathesis your chances of success are greatly lessened. If you find only slight exposure and no pain, protect the pulp from extraneous matter as soon as possible. That is the simple course to be pursued when you find the pulp exposed and in healthy condition.

I have seen failures which have resulted after various modes of treatment. One of my teachers advocated the use of collodion as one of the best possible substances for a cap. He claimed that in its liquid state it fits very perfectly upon the pulp and dries there. Experience has brought to notice serious objections to collodion, however. It appears that in the process of drying it contracts in such a manner as to tend to draw the pulp out of its bed and thus excites inflammation. In a similar manner it may draw loose from the sides of the pulp cavity, perhaps remaining attached at one point, so that we have a moveable cap. I have therefore thought that collodion ought to be abandoned. I formerly tried it prepared by the addition of turpentine, according to an approved formula to avoid shrinkage. I found that in time it decomposed and was a source of trouble. We want something that will simply inclose the pulp, and that will not irritate it by contact. Hill's stopping, warmed and pressed down, will do very well. Notice whether or not the operation is painful. Fill temporarily and await results before venturing to fill with gold. In my own practice I sometimes fail, but do not feel disposed to abandon the method on that account.

A case of exposure of a nerve came under my observation some time ago. The patient was a young lady. She was the perfect picture of health which circumstance I thought was favorable to an attempt to save the pulp. The tooth was a bicuspid. I capped it carefully with a shaving of Hill's stopping. After drying, I filled up the cavity with Hill's stopping. Two weeks ago I removed the filling. The tooth was in its normal condition. There was a little button of secondary dentine protecting the pulp. Through pure awkwardness I chipped that off, exposing the pulp the second time. Hæmorrhage followed. I allayed the pain, washed out the cavity, and repeated the operation of capping. After waiting four days, found there was no pain or uneasiness, removed all the filling, excepting the cap. Filled with gold and expect the tooth to do well.

During the winter of 1864 and 5, I treated a tooth where the nerve was exposed but in healthy condition. Filled it permanently and the case has done well.

I do not claim that I succeed in all cases. I never was absolutely successful in every instance in other matters.

The Dr. then protested against the unnecessary destruction of the pulp. "When you have destroyed the pulp you have virtually destroyed the tooth. Few teeth are firm and strong enough to endure permanently after the pulp is gone. Let me advise you in conclusion, in treating pulps; let all your manipulations be very delicate and careful. We often forget how delicate a structure we have to deal with. Do not over do. Avoid disturbing the pulp. Remove the debris and then cap carefully as soon as possible.

Dr. Rehwinkle: The remarks of Dr. Watt are so well to the point that it would be folly for me to go over them again, it would be a waste of time. I fully concur in what he has said. I will say farther, that I would attempt to save the pulps alive in similar cases, at least in districts where there is no malaria to contend with. I would attempt it even in cases of fever. When the dental pulp is merely exposed, whether it has been done by the encroachment of caries or through accident, and it is otherwise in the performance of its natural functions, there is no reason why it should not be preserved. If the pulp therefore is accidentally wounded so far from feeling discouraged as to the final results of an attempt to save it, it would not disturb me in the least. I would be very careful however what agents I employed. I think in many cases pulps are doctored to death. Creosote has been almost universally used. I ask how many times has it been used intelligently and with discrimination? How many times has it been used when the operator knew just what he wanted to accomplish? Now I should think it perfect folly to put creosote upon a pulp which is in healthy condition. Why? Because it is an escharotic. We would not think of using creosote in its full strength upon an ordinary wound in order to induce it to heal by first intention. We might use it diluted to sponge out the wound. Dentists have been using carbolic acid in the same way, and it has been considered to be all right. We should employ the simplest treatment and the mildest remedies, because in the very elements of the blood we have recuperative power. If we want to go farther and assist the formation of secondary dentine we may employ escharotics. Generally speaking we should employ remedies which soothe the nerve. I do not know that the time has arrived to announce certainly that we have found such a remedy. The lacto-phosphate of lime, it is claimed, promotes the rapid formation of secondary dentine. Dr. Cravens, of Missouri, was the first to introduce this remedy to the attention of the profession in convention a year ago. Since then I think a number of gentlemen have been experimenting with it, with the view of promoting the formation of secondary dentine. I can not myself give very encouraging report of it. Think it is of value in simple and uncomplicated cases. Where I think the saving of a tooth is practicable, I find this remedy goes far toward promoting good results. Some enthusiasts in the profession claim that almost any pulp no matter what its pathological condition, can be saved; that there is scarcely a circumstance under which a pulp may not be saved, even if it be partially devitalized and has sloughed off. I have never been able to accomplish this. I have made most signal failures, simply because of ignoring the circumstances and conditions which were necessary to be observed. Success is sometimes claimed upon insufficient data. I believe that gentlemen who make such favorable statements would not willfully misrepresent. But I think that they have not in all instances come to a correct knowledge of their own cases. A patient goes away and they hear nothing more from him, and since they hear nothing to the contrary take it for granted that all is well. Somebody else may perhaps have given the finishing touch to that pulp afterward. I have had cases of that kind come to my knowledge. I suppose you have. To save a pulp which has been exposed for an indefinite length of time, and has undergone many stages of inflammation where even the greater portion of it has sloughed off, is in my opinion utterly impossible. Whether it be attempted in a malarious district or any other, the result will be the same. It is not practicable in the nature of things, even in surgery itself. If a limb has mortified it must as a general thing be amputated. No surgeon in such a case hesitates to use the knife. In the cases of exposure of the nerve, however, even after inflammation has set in, I think an attempt should be made to save it. As to the remedies to be used in inflammation, we never have come to any definite conclusion. A remedy which answers the purpose in one case may not do so in another. The results depend almost entirely, I believe, upon the judgment of the operator and his ability for justly estimating all conditions, and taking all the accompanying circumstances into consideration, and then proceeding intelligently, and selecting remedies such as are indicated by the peculiarities of the case. In one case, I find chloride of zinc is a valuable astringent to allay inflammation, in another case, I may use aconite, tincture calendula, or arnica. The great secret of success is, not to over do. An inflamed pulp soon suppurates. Sometimes this occurs in a day or two. Success need not be despaired of, if the case be treated in time.

The causes of failure can be summed up as follows: Want of skill in manipulation, want of judgment in selecting remedies, etc., and above all, in doing too much. Lacto-phosphate of lime has proved valuable in my hands, so far as I know, that is to say I know certainly of no cases of failure. Three years ago success was claimed for the use of oxy-chloride of zinc. We have learned however that it exerts a pernicious effect if applied in a semi-fluid state to the pulp. It is a powerful escharotic. Mixed to the consistency of putty it is less objectionable. In many cases where the pulp is supposed to be alive, after an operation you will find upon examination that it is actually dead. I have been deceived in this respect several times. Having had occasion to refill teeth in which the pulp had been capped, in removing the temporary filling and cutting into the dentine, I have remarked the absence of pain or tenderness. The color of the tooth has appeared normal, but the pulp has been dried up, mummified. Open the cavity freely in such a case and in three days you have inflammation of the root-membrane, and no end of trouble. Gentlemen pride themselves on their success, but if they will investigate they will find failure in many cases.

Dr. J. Taft: This subjeet has been discussed so often in our associations that in attempting to talk about it now, we labor under a feeling of restraint and embarrassment, lest we should appear simply to reiterate what has been said before. and especially for the reason that I am aware that others understand the subject as well or better than I do.

Such is the importance of the subject that we can hardly overrate it, It has oftentimes been said, "Oh that subject is worn out, it is hackneyed." I say it is not worn out or hackneyed as long as the results of our investigations are not the best which we can reasonably hope to achieve, and as long as we can discover indications of ultimate success in the reports of experiments and observations which are made year by year, by the many earnest scientific men who are interested in knowing how they can best subserve the interest of their patients. This subject, I repeat, is not by any means threadbare. The best informed among us can gather much that will be available in practice and those who are not so well informed can gather still more. By the discussion of this subject we shall at least be stimulated to still farther investigation and research. There is not one of us who has accomplished all that he can accomplish in the way of experiment and investigation. The final result has not been attained when you say, "I operated upon an exposed pulp after the most approved methods, and the result was failure." That does not prove the fallacy of attempting to save exposed pulps. To study, as Dr. Watt has said, conditions and temperament, and endeavor to learn all about the peculiarities of the case and what the requirements are, and then perform your operation in conformity with those requirements, constitute the elements of success. You hear many say, "I treated a pulp so and so," without even reference to conditions. You may rest assured that he who talks on that wise does not recognize conditions. He has settled into a groove and he runs back and forth continually—never leaving it. When he gives his mode of treatment by successive steps, without making reference to systemic or local conditions, thus indicating that he fails to recognize them, I predict that his operations will eventuate in failure, nine cases out of ten. Many physicians practice medicine in the same way, and the fact that they do not kill all their patients is that the recuperative powers of the patients, who are fortunately able to survive, are so excellent as to resist mal-treatment. The preservation of a pulp is very important, not only as far as the individual tooth is concerned, but as affecting the other teeth as well. If only one tooth becomes enfeebled or diseased, or looses its pulp, the adjacent parts become more or less involved. This varies of course according to the susceptiblities of the parts. Other teeth may be injured by the presence of the tooth, the pulp of which is dead. Irritation of the surrounding parts is far more likely to occur after the pulp is dead. The devitalized tooth deteriorates with varying rapidity, and sooner or later will break away and be lost, and of course the effect upon the other teeth is injurious. These facts are palpable to every one who has studied the matter and is familiar with the subject. Pulps of teeth frequently become devitalized before they are exposed at all. They sometimes become devitalized—such is the susceptibility to disease —before the tooth is even decayed. Others become exposed and irritated, inflammation sets in, and the case runs through the various phases of disease, and in spite of adverse circumstances they survive. These facts demonstrate that there is a great variety of systemic conditions. The pulp is more amenable to treatment by recognizing and dealing intelligently with these conditions. How few ever think of systemic conditions or regulate their practice by considerations arising from general pathological symptoms. We find a pulp exposed and our usual treatment is simply of a local character it is seldom directed to an honest endeavor to change the general systemic conditions to a more favorable state. But you ask, "what can I do?" That is just the question. We can not here have a course of lectures, three to six months long, to discuss the principles involved. I think it important that all should study the subject well, and learn from personal observation that which can be learned in no other way. Now what are some of the conditions which would modify treatment of the pulp? Suppose the blood is in an impoverished condition. It is not to be expected in such a case that you can control the lesion with the same facility as if the blood were in good healthy condition. Supposing the blood is deficient in some important and indispensable constituent, perhaps the patient is anæmic, or there is a deficiency of fibrine, or red or white corpuscles of the blood; all these these things would operate as impediments. The corpuscles may be defective and so be unable to perferm their proper function. The influence of these conditions will extend to the pulp, as to the eye or other organ of the body. Perhaps some insidious poison is at work. You know how difficult it is to treat successfully a case where there is malarial influence. If you were going to select cases for treatment you would certainly avoid those which you know were charged with malaria. Change these unfavorable conditions, or if you are incapable, refer the case to some one who can. Here we see the necessity of the dentist's knowledge, embracing the whole range of medical science, and acquainting himself with the more minute principles of the science. We find different susceptibilities in cases which are apparently exactly similar. Do not make a blind dash at such cases. You find two persons who are apparently alike in condition. The one is far more susceptible to a given course of treatment than the other. Teeth, too, differ in their organization. At one period in life a tooth is more dense in its structure than at another.

